# Sex, Drugs, and TBI: The Role of Sex in Substance Abuse Related to Traumatic Brain Injuries

**DOI:** 10.3389/fneur.2020.546775

**Published:** 2020-10-19

**Authors:** Robin Oliverio, Kate Karelina, Zachary M. Weil

**Affiliations:** Department of Neuroscience and Rockefeller Neuroscience Institute, West Virginia University School of Medicine, Morgantown, WV, United States

**Keywords:** traumatic brain injury, substance abuse, sex differences, epidemiology, adolescent brain injury

## Abstract

Traumatic brain injuries (TBI) are a significant public health problem costing billions of dollars in healthcare costs and lost productivity while simultaneously reducing the quality of life for both patients and caregivers. Substance abuse is closely interconnected with TBI, as intoxicated individuals are at a greater risk of suffering brain injuries, and TBI may serve as a risk factor for the subsequent development of substance use disorders. There are also prominent sex differences in the etiology, epidemiology, and consequences of TBI. For instance, men are more likely to be injured on sporting fields or in auto accidents, while women are disproportionately likely to suffer TBI associated with intimate partner violence. Moreover, while men are much more likely to suffer TBI during late adolescence–young adulthood, sex differences in the incidence of TBI are much less prominent during other developmental epochs. Further, there are prominent sex differences in substance abuse biology; for example, while more men meet diagnostic criteria for substance abuse disorders, women tend to advance from casual use to addiction more quickly. In this paper, we will discuss the emerging clinical and preclinical evidence that these sex differences in TBI and substance abuse interact and may be prominent determinates of long-term outcomes.

## Introduction

Sex differences are prominent components of the biology of substance abuse. Men more commonly partake in substance use and are more likely to develop dependence than women (1–4), although compared to men, women tend to more rapidly progress from beginning use, to dependence, to treatment-seeking of many substances including alcohol, marijuana, and cocaine (2, 5, 6). This difference is commonly observed in alcohol use disorder (AUD), where women often begin drinking at a later age and progress through the stages of abuse at a faster rate than men (6, 7). However, this concept has become contentious, with recent studies suggesting that this phenomenon is not the case in the general population and that sex differences in AUD have begun to decrease (4, 8).

Sex differences exist within the withdrawal stage of substance abuse as well. Compared to men, women have shorter periods of cessation from smoking and report greater difficulties in quitting (2, 9, 10). Hogle and Curtin (11) found that women showed more of a negative affective response to a conditioned fear stimulus during nicotine withdrawal. Additionally, women attempting to quit cocaine have shorter periods of abstinence than men and report more intense cravings in response to stimuli related to cocaine use during withdrawal (12, 13).

Here, we will examine the emerging data on sex differences in substance abuse among traumatic brain injury (TBI) survivors. TBI places a significant burden on health and the economy in the United States, causing ~1.7 million individuals to seek treatment and costing $60 billion in combined indirect and direct costs annually (14, 15). Further, TBI often results in long-term disability, leading to an increased burden on relatives and substantial decreases in lifetime productivity (16, 17). Sex differences in TBI incidence are well-documented; however, given the intense media attention on the role of contact sports-related head injuries, and the general focus on men across biomedical research, it is not surprising that disproportionate research attention has been focused on men. This approach is very likely to undermine progress in prevention and treatment of TBI and related outcomes in both sexes. Putting aside for a moment the possibility that TBI pathophysiology may be different across sexes, it is absolutely clear that there are prominent sex differences in TBI incidence and etiology. For instance, men are more commonly treated than women for TBI (14), although this sex difference in the incidence of TBI may well-underestimate the true scope of injuries to women, as men tend to suffer more severe injuries and are consequently more likely to seek (or require) medical treatment. Critically, this prominent sex difference in TBI incidence narrows considerably if stratified across ages. The largest male bias in TBI incidence occurs between adolescence and young adulthood and is largely absent in other developmental epochs (most prominently early childhood and retirement age). Moreover, the etiology of injuries also differs prominently. For example, men are much more likely to suffer a TBI in the workplace, or on the sporting field, while women are disproportionately injured by intimate partner violence (18–20). Epidemiological results vary with respect to sex differences in outcomes following TBI, although some studies suggest that women fare worse in a majority of measurements, particularly for mild injuries, whereas men appear to exhibit worse outcomes after more severe injuries (21–25). Women experience a longer duration of posttraumatic amnesia and hospitalization and have a greater likelihood of requiring surgical intervention (21, 24). Female TBI patients are also more likely to be admitted to the intensive care unit (ICU) and remain in the ICU longer than male patients (26). Additionally, among mild TBI patients, women are more likely to experience long-term (3 years postinjury) postconcussion symptoms than men, including headache, dizziness, nausea, noise sensitivity, fatigue, sleep disturbance, and spinal pain (27). Adult women who sustained a pediatric TBI more commonly report symptoms consistent with major depressive disorder and anxiety disorders compared to adult men who report more externalizing behavior such as aggression and substance abuse (28). Moreover, even among the adolescent–young adult cohort, the disparity between injury incidences among the sexes might be decreasing due to an increasing prevalence of female involvement in sports and military combat (29–31).

**Figure 1:**
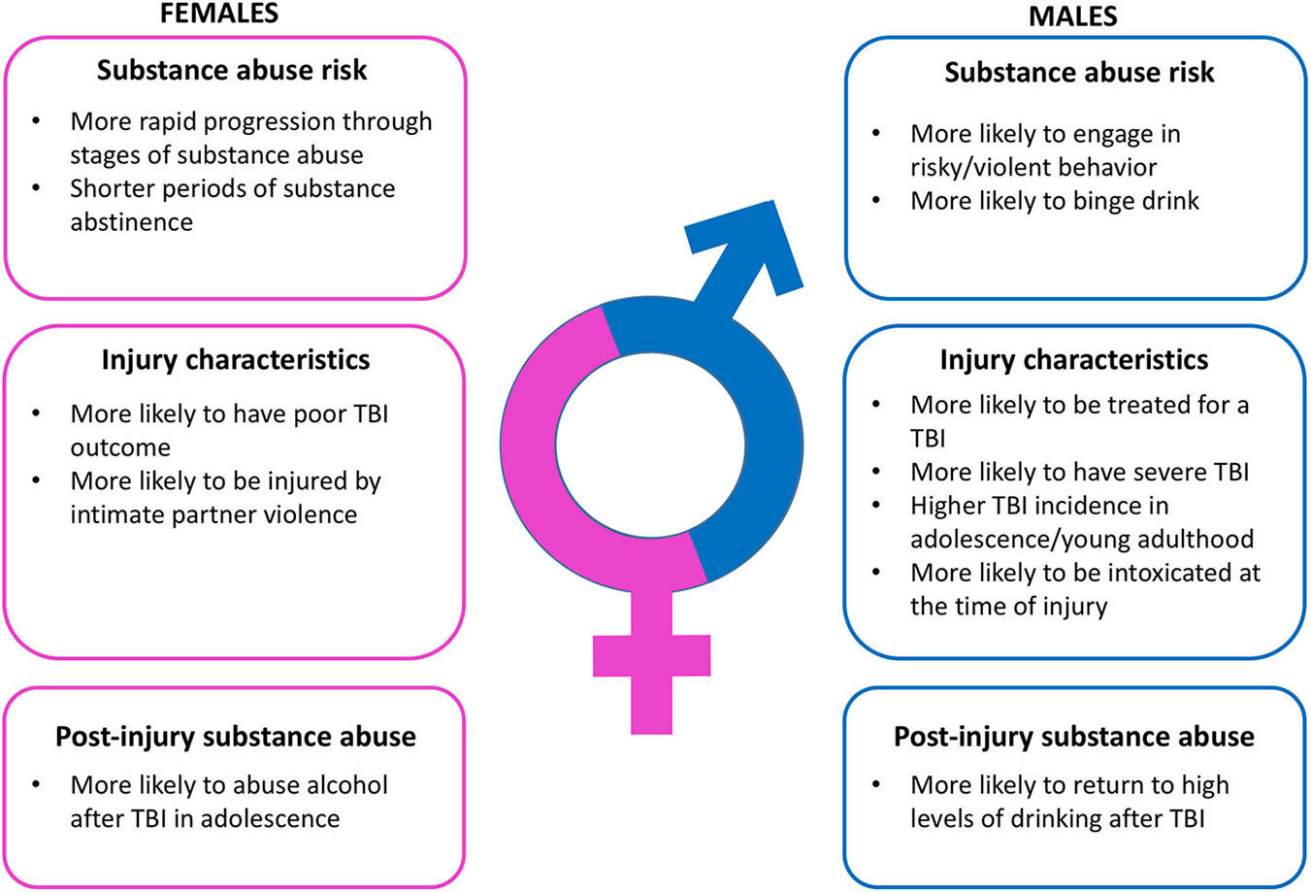
Summary of relevant sex differences in substance abuse risk, traumatic brain injury characteristics, and post-injury substance abuse.

These sex differences in incidence, severity, and etiology are critically important determinants of the relationship between TBI and substance abuse (Figure 1). TBI is bidirectionally linked to substance abuse. First, intoxication at the time of injury is a very common feature of TBI patients (32, 33), and driving while intoxicated has been noted to increase the risk of TBI (34, 35). The impact of intoxication while driving on TBI outcomes varies considerably, with some studies indicating that high blood alcohol level during a motor vehicle accident increases TBI severity (36, 37), some providing evidence of decreased TBI severity (38–40), and still others showing no significant difference in TBI outcome (41, 42). Of note, the prevalence of intoxication at the time of injury can vary significantly across sex. For instance, among TBI patients treated at one of two trauma centers in the Netherlands, 33% of individuals were intoxicated at the time of their injury. This subset of patients was both younger (38 years of age) and more likely to be male (78%) than the patients who were not intoxicated at the time of their injuries (40 years of age and 60% male) (43). The larger number of men intoxicated at the time of their injury is not entirely surprising given that younger men are more likely to engage in risky or violent behavior and that this is exacerbated by substance use (44, 45).

One consequence of the large number of individuals intoxicated at the time of their injuries is that the TBI patient population consists disproportionately of individuals with a history of substance misuse (46). This is important because a history of substance abuse, as well as continued use after injury, can predict worsened outcomes, reduced recovery, and even increased likelihood of subsequent TBIs (38, 47). For example, a history of substance abuse increases the probability of a more severe injury from motor vehicle accidents or falls from great heights (39), as well as a greater probability of mass lesions and mortality and poorer outcomes upon release from the hospital (38). Additionally, a history of alcohol abuse prior to brain injury is associated with greater neuropsychological deficits and mood disorders following a TBI (47, 48). In general, among individuals with a history of alcohol abuse, drinking behavior tends to decline acutely after injury, particularly with more severe injuries (49). However, some percentage of individuals (more commonly men) gradually return to high levels of drinking after their injuries (50, 51).

Third, experiencing a TBI, especially prior to or during adolescence, is associated with a greater risk of developing a substance use disorder later (52–54). This relationship has been difficult to establish epidemiologically; however, there is mounting evidence that early TBI can serve as a risk factor for the development of substance abuse issues [reviewed in (55)]. There is much less known as to whether these relationships are similar across sexes, and the potential roles are often overshadowed by the higher baseline levels of both TBI and substance abuse among men (56). One interesting finding that we recently reported emerged from an analysis of individuals from Ohio self-reporting their history of TBI and current drinking patterns. Individuals with a history of TBI before age 20 were more likely to binge drink as adults, and regardless of injury history, men reported higher incidence of binge drinking than did women. However, women injured during adolescence were more likely to drink than those injured *either* early or later in their lives, an effect that was not apparent in men (57). These data indicate that sex differences in the patterns and types of injuries could have major implications for the relationship between TBI and substance abuse.

Given that there are clear sex differences in substance abuse and TBI, it follows that sex differences present differential risks for the development of substance abuse disorders following TBI. However, this remains largely unstudied. Epidemiological studies in veterans who experienced a TBI show that men are more likely than women to develop an alcohol use disorder (AUD) and non-alcohol substance use disorder (SUD) as well as generally exhibit alcohol misuse (58, 59). The overall prevalence of alcohol misuse after TBI is 6.8–16.2% in women compared with 20.3–27% in men. Further, men who sustained a mild TBI prior to adulthood are more likely to report experiencing substance abuse and dependence (28). However, overall sex differences in substance abuse following TBI remain poorly understood and severely understudied. Much more investigation is needed in this area to elucidate this relationship.

Preclinical studies into the relationship between TBI and the development of substance abuse issues have reported that TBI can facilitate drug-related behavior. Specifically, TBI has been shown to enhance self-administration or conditioned place preference acquisition of alcohol (60, 61), cocaine (62, 63), and opiates (64) although not all studies have reported facilitating effects of injury on drug-related behavior [e.g., (65)]. Notably, virtually all these studies were conducted in male rodents. One study that did systematically examine potential sex differences reported that female, but not male mice, injured at postnatal day 21 exhibited enhanced alcohol self-administration (61). This appeared to be due to differences in the rewarding properties of alcohol, as immediate early gene activation was altered in the reward pathway following alcohol injection and conditioned place preference responses to alcohol were apparent in injured female mice only. Moreover, injury in adulthood did not alter alcohol-related behavior in either sex. Thus, much like in the clinical picture, sex and age at injury are critical determinates of substance-abuse-related phenomena (57, 66).

## Conclusion

Our understanding of the patterns and consequences of TBI are rapidly evolving, and it is becoming increasingly clear that men and women vary significantly in the incidence and consequence of these injuries. Moreover, TBI-related substance abuse is a major issue that can significantly alter long-term outcomes and the risk for repeated injuries. We know that there are prominent sex differences in substance abuse more generally, and thus, it is highly likely that sex will be a critical determinant of the relationship between TBI and substance abuse, although much more clinical and preclinical work is necessary.

## Author Contributions

RO, KK, and ZW wrote and edited the manuscript. All authors contributed to the article and approved the submitted version.

## Conflict of Interest

The authors declare that the research was conducted in the absence of any commercial or financial relationships that could be construed as a potential conflict of interest.
